# Development of Duplex LAMP Technique for Detection of Porcine Epidemic Diarrhea Virus (PEDV) and Porcine Circovirus Type 2 (PCV 2)

**DOI:** 10.3390/cimb44110368

**Published:** 2022-11-03

**Authors:** Supatra Areekit, Pongbun Tangjitrungrot, Sintawee Khuchareontaworn, Kankanit Rattanathanawan, Pornpun Jaratsing, Montri Yasawong, Gaysorn Chansiri, Nareerat Viseshakul, Kosum Chansiri

**Affiliations:** 1 Innovative Learning Center, Srinakharinwirot University, Bangkok 10110, Thailand; 2 Center of Excellence in Biosensors, Srinakharinwirot University, Bangkok 10110, Thailand; 3 Chulabhorn Graduate Institute, Chulabhorn Royal Academy, Bangkok 10210, Thailand; 4 Department of Industrial Pharmacy, Faculty of Pharmacy, Silpakorn University, Nakhon Pathom 73000, Thailand; 5 Parasitology Unit, Department of Pathology, Faculty of Veterinary Science, Chulalongkorn University, Bangkok 10330, Thailand

**Keywords:** duplex loop-mediated isothermal amplification, duplex LAMP, porcine epidemic diarrhea virus, porcine circovirus type 2

## Abstract

Porcine epidemic diarrhea virus (PEDV) and porcine circovirus type 2 (PCV2) are both important global pathogenic viruses which have a significant impact on the swine industry. In this study, a duplex loop-mediated isothermal amplification (duplex LAMP) method was developed in combination with lateral flow dipstick (LFD) for simultaneous detection of PEDV and PCV2 using specific sets of primers and probes designed based on the conserved regions of a spike gene (KF272920) and an ORF gene (EF493839), respectively. The limit of detection (LOD) values of the duplex LAMP-LFD for the detection of PEDV and PCV2 were 0.1 ng/µL and 0.246 ng/µL, respectively. The LOD of duplex LAMP-LFD was 10-times more sensitive than conventional PCR and RT-PCR-agarose gel-electrophoresis (PCR-AGE and RT-PCR-AGE). No cross-reaction to each other and to other pathogenic viruses that can infect pigs were observed according to analytical specificity tests. The duplex LAMP-LFD method for the simultaneous detection of PEDV and PCV2 co-infection could be completed within approximately 1.5 h, and only a simple heating block was required for isothermal amplification. The preliminary validation using 50 swine clinical samples with positive and negative PEDV and/or PCV2 revealed that the sensitivity, specificity, and accuracy of duplex LAMP-LFD were all 100% in comparison to conventional PCR and RT-PCR. Hence, this study suggests that duplex LAMP-LFD is a promising tool for the early detection and initial screening of PEDV and PCV2, which could be beneficial for prevention, planning, and epidemiological surveys of these diseases.

## 1. Introduction

Porcine epidemic diarrhea virus (PEDV) and porcine circovirus type 2 (PCV2) are both important global viruses with a significant impact on the swine industry. The PEDV has been endemic in most parts of the world since its emergence in the 1970s [1]. In swine of all ages, the symptoms of infection are watery diarrhea and vomiting, and suckling piglets have a mortality rate of up to nearly 100%. The majority of PEDV transmission occurs via the fecal–oral route. However, airborne transmission via the fecal–nasal route from pig-to-pig and farm-to-farm are also important for the spread of virus [2]. Pathogenic strains of PEDV have become pandemic due to pathogenic PEDV outbreaks in Japan, Korea, Thailand, the Philippines, the Western hemisphere and, subsequently, Portugal and Germany [3]. Theoretically, PEDV has a single-stranded positive-sense RNA genome of approximately 28 kb in size encoding for four structural proteins (spike (S), envelope (E), membrane (M), and nucleocapsid (N)), including four nonstructural proteins (1a, 1b, 3a, and 3b) [4]. Among these, the S protein is crucial for viral entry via interactions with specific glycoprotein receptors on the host cell surface, inducing neutralizing antibodies [5]. Phylogenetic analysis based on the PEDV spike gene has been widely determined and has revealed that it consists of conserved, semi-conserved and hypervariable regions, which are informative for genetic variation of the virus [6]. Among them, the conserved regions on the spike gene have been targets for the design of specific universal primers and probes in the development of molecular detection of PEDV isolates. In 2022, Li and coworkers published a systematic review and meta-analysis of the diagnostic accuracy of lateral flow immunochromatography assay (ICA)-based procedures and nucleic acid isothermal amplification (NAIA)-based point-of-care tests (POCTs) for detecting PEDV [7]. Their study revealed that NAIA-based POCTs had a better diagnostic performance than ICA-based POCTs, and both tests showed acceptable diagnostic accuracy.

Aside from PEDV, the postweaning multisystemic wasting syndrome (PMWS) caused by porcine circovirus (PCV) also has a large impact on the swine industry [3]. Among various PCV strains, the pathogenic PCV2 has been widespread in pig populations for decades, but began to severely affect pig production worldwide in the late 1990s [8]. Clinically, the manifestation of the disease is progressive weight loss, respiratory signs (tachypnea, dyspnea) and jaundice [9,10]. Transmission of PCV2 occurs horizontally (pig-to-pig contact) and vertically (sow-to-piglet) via direct contact with contaminated respiratory, digestive, and urinary secretions, via virus-contaminated feed, or via sexual contact, or artificial insemination [8,11,12]. Additionally, wild boars are also susceptible to PCV2 infection through migration or trade of contaminated feed, or via sub-clinically infected pigs [13]. When a new genotype of PCV2 enters a country, it can transfer between domestic pigs and wild boar populations, and thus wild boars may act as both a reservoir and a vector for PCV2.

According to epidemiologic data, PCV2 can be classified into several genotypes; the main global genotypes are PCV2a and PCV2b, which are generally related to severe disease [8]. PCV2 is a non-enveloped virus that contains a single stranded circular DNA genome of 1.7 kb [14]. The genome of PCV2 is predicted to contain 11 open reading frames (ORFs), including ORF1 and ORF2 encoded for replicase (Rep and Rep’) and capsid (Cap; also, Cp) protein, respectively. At least eight genotypes of PCV2 are further recognized (a–h) based on ORF2 [15]. In addition, at least four other functional ORFs in the PCV2 genome (ORF3, ORF4, ORF5, ORF6) have been identified [16]. At present, PCV detection methods mainly rely on either nucleic acids or serum antibodies of this pathogen, including virus isolation, electron microscopy, immunofluorescence assay (IFA), immunohistochemistry (IHC), fluorescence in situ hybridization (FISH), enzyme-linked immunosorbent assay (ELISA), polymerase chain reaction (PCR), real-time fluorescence quantitative PCR (qPCR), and loop-mediated isothermal amplification (LAMP) [17,18,19,20,21,22,23,24]. The selection of a diagnostic method for detection of PCV2 depends on many factors such as sensitivity, specificity, on-site utility (portability), equipment or laboratory requirements, professional skill, complexity, cost per detection, efficacy and process time. In the case of co-infection of PCV2 with other viruses such as PEDV, a suitable diagnosis method with high sensitivity and specificity are necessary.

Currently, the co-infection of PEDV and PCV2 causes high economic losses in the swine industry worldwide, of which 29.9 percent naturally occurs [25,26]. In Thailand, co-infection of PEDV and PCV2 in swine has been reported and caused more severe clinical symptoms than a single infection. Previous studies have indicated that multiplex conventional RT-PCR could detect and differentiate the co-infection of swine PEDV and PCV. However, the disadvantage of multiplex RT-PCR assay is that it is time-consuming and requires expensive PCR cyclers [27]. Furthermore, the PCR products need to be monitored and analyzed using gel-electrophoresis (AGE). In this article, a duplex loop-mediated isothermal amplification (duplex LAMP) method combined with lateral flow dipstick (LFD) was developed for simultaneous detection of PEDV and PCV2 in swine clinical samples. The results are observed from the appearance of purple color at the test line of the strip. The limit of detection (LOD) and analytical specificity of the assay were compared against conventional PCR-based methods. The duplex LAMP-LFD underwent preliminary validation using 50 swine clinical samples (16 samples of PEDV, 14 samples of PCV and 20 negative controls) and % accuracy, % diagnostic sensitivity, % diagnostic specificity of the assay were calculated in comparison to PCR-based methods.

## 2. Materials and Methods

### 2.1. Samples and Nucleic Acid Extraction

The swine PEDV RNAs and PCV DNAs were provided by Animal Health Diagnostic Center, Bangkok, Thailand. Briefly, nucleic acid extraction was achieved using Viral Nucleic acid extraction kit II (Geneaid, New Taipei, Taiwan) according to the manufacturer’s instructions. Then, the PEDV RNAs were subsequently transcribed to cDNA by the addition of 5× reverse transcriptase supermix for RT-qPCR (Bio-rad, Hercules, CA, USA) and were incubated at 37 °C for 20 min. The PCV2 genomic DNA and the PEDV cDNA were dissolved in sterile distilled water prior to DNA amplification. The DNA concentration was determined using a NanoDrop™ 2000 Spectrophotometer (Thermo Scientific, Wilmington, DE, USA).

### 2.2. Design of Probes and Primers

**Figure 1:**
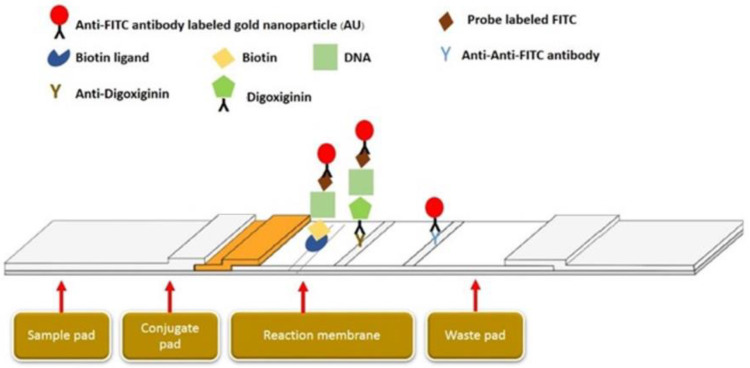
Schematic of LFD assays.

The amplification methods were performed using specific primers and DNA probes designed based on the spike gene of PEDV (KF272920) and the ORF gene of PCV 2 (EF493839). The Primer explorer version 5 (http://primerexplorer.jp/lampv5e/index.html) (accessed on 5 January 2020). program was used to check some essential characteristics of the probes and primer sequences, such as hair-pin loop, self-binding, and melting temperature (Table 1). According to the LAMP primers, the FIPs of PCV2 and PEDV were labeled as biotin and digoxiginin, respectively. The DNA-specific probes were tagged with carboxyfluorescein (FITC) at the 5′-end of the sequences for detection of the LAMP products via biotin/biotin ligand and digoxiginin/anti-digoxiginin on the two different test lines. The positive purple color on two test lines developed upon the formation of the biotin ligand/biotin-PCV2-LAMP amplicon/PCV2-DNA probe-FITC/anti-FITC-gold nanoparticles complex and the anti-digoxiginin/digoxigenin-PEDV-LAMP amplicon/PEDV DNA probe-FITC/anti-FITC-gold nanoparticles complex (Figure 1).

### 2.3. PCR-AGE

Each PCR amplification of PCV2 and PEDV was prepared in 25 µL of reaction containing 1 µL of each DNA target, 10× PCR buffer, MgCl_2_, 10 mM dNTPs, 5 units of Taq polymerase (New England Biolabs, Ipswich, MA, USA), and a 10 µM concentration of each primer. The PCR was performed using a C1000 Touch™ Thermal Cycler (Bio-Rad Laboratories Ltd.; Hercules, CA, USA) with a pre-denaturation step at 94 °C for 5 min followed by 30 cycles of denaturation at 94 °C for 1 min, annealing at 50 °C for 1 min and extension at 72 °C for 1 min. After that, the reaction was terminated via post-extension at 72 °C for another 5 min prior to analysis using 2% agarose gel electrophoresis (AGE) in 0.5× Tris/Borate/EDTA (TBE) buffer at 100 volts. The DNA pattern was observed under UV light using gel-doc (UVITEC Cambridge, Cambridge, UK).

### 2.4. LAMP-AGE

Each LAMP amplification of PCV2 and PEDV was prepared in 25 µL reaction containing 1 µL of each cDNA target, 10× LAMP buffer, Betaine, 25 mM dNTP, MgSO_4_, 8 units Bst DNA polymerase (New England Biolabs, Ipswich, MA, USA), 10 µM of each outer primer (PCV2-F3/PCV2-B3 and PEDV-F3/PEDV-B3), 100 µM of each inner primer (PCV2-FIP/PCV2-BIP and PEDV-FIP/PEDV-BIP), and 10 µM of each probe (PCV2-probe and PEDV-probe). The reaction mixture was incubated at 63 °C for 60 min using the C1000 Touch™ Thermal Cycler (Bio-Rad Laboratories Ltd.; Hercules, CA, USA) and was analyzed using 2% agarose gel electrophoresis in a 0.5× Tris/Borate/EDTA (TBE) buffer at 100 volts. The DNA pattern was observed under UV light using gel-doc (UVITEC Cambridge, Cambridge, UK).

### 2.5. Duplex LAMP-LFD

The duplex LAMP amplification of PCV2 and PEDV was prepared in 25 µL reaction containing 1 µL of each DNA target, 10X LAMP buffer, Betaine, 25 mM dNTP, MgSO_4_, 8 unit Bst DNA polymerase (New England Biolabs, Ipswich, MA, USA), 10 µM of outer primers (PCV2-F3/PCV2-B3 and PEDV-F3/PEDV-B3), 100 µM of inner primers (PCV2-FIP/PCV2-BIP and PEDV-FIP/PEDV-BIP), and 10 µM of PCV2-probe and PEDV-probe. The reaction mixture was incubated at 63 °C for 60 min using a C1000 Touch™ Thermal Cycler (Bio-Rad Laboratories Ltd.; Hercules, CA, USA)

After duplex LAMP, 5 µL of the duplex LAMP amplicon mixture was transferred to a new microcentrifuge tube and 100 µL of the assay buffer containing Tris-buffered saline was added. Finally, the commercial 2-test line LFD strip (Milenia Biotec GmbH, Gießen, Germany) was dipped in the mixture and left for 5–10 min prior to dipping into water for another 10 min to stop the reaction.

### 2.6. LOD and Analytical Specificity Tests

To determine the LODs of all the tests, 10-fold serial dilutions of PCV2 DNA and PEDV cDNA were manipulated in the range of 2.46 fg to 24.60 ng and 1.00 fg to 10.00 ng, respectively.

The analytical specificity test of duplex LAMP-LFD was tested against porcine epidemic diarrhea virus (PEDV), porcine circovirus (PCV), Aujeszky’s disease virus (ADV), foot and mouth disease virus (FMDV), transmissible gastroenteritis virus (TGEV), porcine reproductive and respiratory syndrome virus (PRRSV EU-strain), porcine reproductive and respiratory syndrome virus (PRRSV US-strain), classical swine fever virus (CSFV) and swine influenza virus (SIV), which were used as templates for evaluation of the specificities of the method.

### 2.7. Swine Clinical Specimens

The 50 swine clinical specimens were gifted from Animal Health and Technical Service Office CPF (Thailand) Public Company Limited with ethical approval. Among them, 16, 14 and 20 samples were identified and confirmed as PCV2-positive, PEDV-positive and negative, respectively, using conventional PCR-based amplification followed by agarose gel electrophoresis analysis. The preliminary validation in terms of % diagnostic sensitivity, % diagnostic specificity, and % accuracy were calculated as indicated in Table 2.

## 3. Results

### 3.1. Optimization of PCR-Based AGE, LAMP-Based AGE and Duplex LAMP-LFD

**Figure 2:**
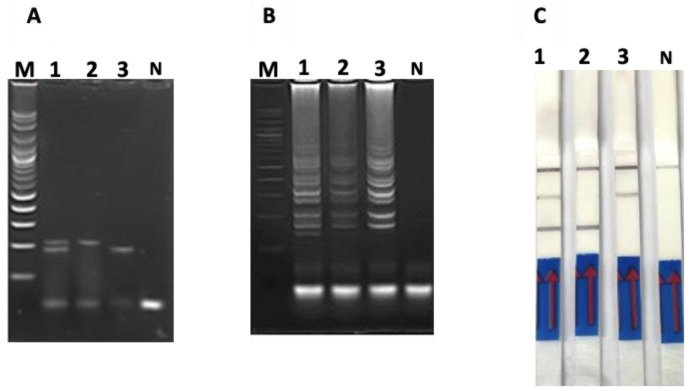
DNA amplification profiles of PEDV spike gene, PCV2 ORF gene, and PEDV/PCV2 co-infection using (**A**) PCR-based AGE, (**B**) LAMP-based AGE and (**C**) duplex LAMP-LFD. Lane M represents 100 bp DNA ladder marker; Lane 1 represents duplex PEDV/PCV2; Lane 2 represents PCV2; Lane 3 represents PEDV; Lane N represents negative control (without DNA template).

Under optimal annealing temperatures, the RT-PCR product of the PEDV spike gene and the PCR product of the PCV2 ORF gene were 187 and 216 bp in size, respectively (Figure 2A). Additionally, no non-specific products were observed with negative controls (Figure 2A).

Upon duplex LAMP of PEDV/PCV2, an amplicon pattern was obtained and visualized on agarose gel electrophoresis (Figure 2B) along with two positive test lines on the LFD strip (Figure 2C). Similarly, RT-LAMP of the PEDV spike gene and LAMP of the PCV2 ORF gene displayed the traditional ladder pattern of LAMP products on agarose gel electrophoresis (Figure 2B) and appeared as one positive test line on each LFD strip (Figure 2C).

### 3.2. LODs of PCR-Based and LAMP-Based Assays

**Figure 3:**
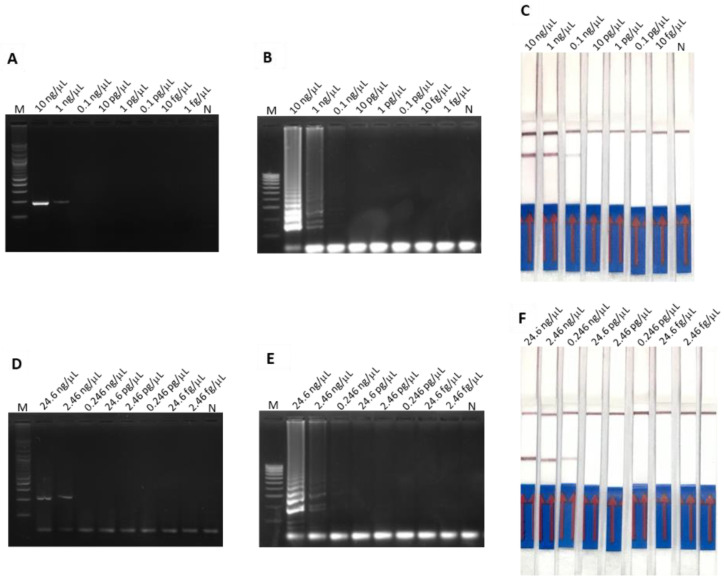
The LOD of PEDV spike gene amplification determined using (**A**) RT-PCR-AGE, (**B**) RT-LAMP-AGE and (**C**) RT-LAMP-LFD. The LOD of PCV2 ORF gene amplification examined using (**D**) PCR-AGE, (**E**) LAMP-AGE and (**F**) LAMP-LFD.

The LODs of both RT-PCR-AGE and RT-LAMP-AGE for detection of PEDV spike gene were 1 ng/µL (Figure 3A,B), whereas those of PCR-AGE and LAMP-AGE for detection of PCV2 ORF gene were 2.46 ng/µL (Figure 3D,E).

The LOD of duplex LAMP-LFD for detection of PEDV spike gene and PCV2 ORF gene were 0.1 ng/µL (Figure 3C) and 0.246 ng/µL (Figure 3F), respectively.

### 3.3. The Analytical Specificity Tests of PCR-Based and LAMP-Based Assays

**Figure 4:**
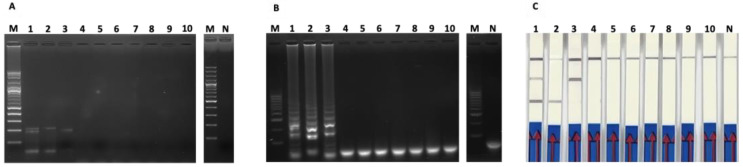
The analytical specificity tests for detection of PEDV spike gene and PCV2 ORF gene using (**A**) duplex PCR-based AGE, (**B**) duplex LAMP-based AGE and (**C**) duplex LAMP-LFD. Lane M represents 100 bp DNA ladder marker; Lane 1 represents duplex PEDV/PCV2; Lane 2 represents PCV2; Lane 3 represents PEDV; Lanes 4–10 represent Aujeszky’s disease virus (ADV), foot and mouth disease virus (FMDV), transmissible gastroenteritis virus (TGEV), porcine reproductive and respiratory syndrome virus European strain (PRRSV-EU), and porcine reproductive and respiratory syndrome virus US strain (PRRSV-US), respectively.

The analytical specificity tests of duplex PCR-based AGE (Figure 3A), duplex LAMP-based AGE (Figure 4B) and duplex-LAMP-LFD (Figure 4C) for the detection of PEDV spike gene and PCV2 ORF gene showed no cross-amplification against Aujeszky’s disease virus (ADV), foot and mouth disease virus (FMDV), transmissible gastroenteritis virus (TGEV), porcine reproductive and respiratory syndrome virus European strain (PRRSV-EU), or porcine reproductive and respiratory syndrome virus US strain (PRRSV-US).

### 3.4. Application on Clinical Samples

Preliminary validation of duplex LAMP-LFD in comparison to PCR-based AGE and LAMP-based AGE revealed that specificity, sensitivity, and accuracy were all 100% (Table 2). The overall process times of PCR-AGE, LAMP-AGE and RT-LAMP-LFD were 2.5, 2.0 and 1.5 h, respectively (Table 3).

## 4. Discussion

In this study, a prototype for a duplex LAMP-LFD assay relevant to Technology Readiness Level 3 (TRL 3) was developed for simultaneous detection of PEDV and PCV 2 in the same specimens. According to the LOD, the duplex LAMP-LFD was 10-times more sensitive than PCR-based AGE and LAMP-based AGE, which correlates with previous reports [28,29,30,31,32,33,34,35,36,37,38,39,40,41,42]. The high analytical specificity of the duplex LAMP-LFD resulted from primers and DNA probes that were uniquely designed from the conserved regions on the spike and ORF genes for PEDV and PCV2, respectively.

As stated in Table 4, the LOD, analytical specificity, % diagnostic sensitivity and % diagnostic specificity of duplex LAMP-LFD were comparable to those of other molecular diagnostic tests. Generally, the fewer viral copies detected, the better the assay. However, if any assays exhibit very sensitive LODs, a false positive signal may be the cause. Additionally, the discrimination between alive and dead viruses is the limitation of molecular assays based on DNA and RNA detection, as a positive result may be generated from the amplification of degraded DNA/RNA fragments of dead virus. As such, clinical investigation is still required to confirm infection. Nonetheless, a rapid, sensitive, and specific assay is still necessary for management of these diseases, especially for the prevention of the viral spread from pig-to-pig and farm-to-farm. Although some of the molecular assays listed in Table 4 present remarkable LODs, expensive equipment and/or complicated protocols are still involved. Due to its equivalent sensitivity and analytical specificity, the duplex LAMP-LFD method is more practical than other molecular tests since it only requires a simple heating block with a less complicated operation. Considering the LOD and analytical specificity, the duplex LAMP-LFD method can be further improved and applied for early detection or initial screenings, which is beneficial for prevention of virus spread in farms and nearby. Nevertheless, the duplex LAMP-LFD assay was unable to differentiate between the genetic diversity of the virus because the primers and DNA probes were created from conserved regions of the PCV2 ORF gene and the PEDV spike gene.

Normally, the bottleneck of any molecular diagnosis is the treatment of specimens using DNA or RNA extraction to eliminate or clean up textures and other components that can interfere with DNA amplification. Hence, a genomic extraction step is essential for molecular assays to reach the best LOD. The development of rapid and easy point-of-care test kits based on direct in situ or one-step detection of clinical specimens is very challenging. We performed a direct DNA amplification of clinical samples without genetic extraction, but low LOD occurred due to the viscous texture (data not shown). Currently, the treatment of specimens without genomic extraction for direct DNA amplification is under investigation in our laboratory.

In this study, the one-step DNA amplification and DNA amplicon–DNA probe hybridization was accomplished to diminish contamination during the pipetting steps in the protocol as well as to be convenient for users. This also shortens the process time of the test so that it can be completed within 60 min after genomic extraction with and without reverse transcription. This suggests that the assay could be further improved and turned into a convenient screening test. The process time of detection is quite important in these situations and a rapid diagnosis test is crucial for disease control and treatment of the infected swine. Therefore, a one-step incubation that can be completed in less than 60 min with an acceptable LOD should be further evaluated.

Considering this preliminary validation in terms of diagnostic sensitivity, diagnostic specificity, accuracy, and coefficient of variation using 50 swine clinical specimens, the duplex LAMP-LFD was comparable to conventional PCR-based AGE, but the overall detection process time was reduced to approximately 1.5 h. To verify the analytical efficacy of this duplex LAMP-LFD assay, more samples should be further investigated and improved to fulfill the point-of-care screening test criteria.

## 5. Conclusions

In conclusion, a duplex LAMP-LFD method was developed for the detection of the co-infected PEDV and PCV2 using specific primers and DNA probes designed based on the spike gene and ORF gene, respectively. The LOD of the test was 10-times better than conventional PCR-based AGE and had 100% diagnostic sensitivity, diagnostic specificity and accuracy. The assay could be completed within 1.5 h and only required a heating block for one-step DNA amplification and hybridization. This assay could be applicable for early detection or initial screening of the viruses, which is beneficial for prevention of virus spread in farms.

## Figures and Tables

**Table 1:** Primers and probes used in this study.

Primers	Sequence (5′ –> 3′)	5′ Conjugated
PCV2_F3	GCTGGCTGAACTTTTGAAAG	-
PCV2_B3	AGCCAGCCATAAAAGTCA	-
PCV2_FIP	GCTTTTACCACACCCAGGTGTTTTTGAGCGGGAAAATGCAGAA	Biotin
PCV2_BIP	GACCCGGAAACCACATACTGGTTTTTTCAATAACAACCACCACTTCTTCAC	-
PEDV-F3	CTTGAAGGTGTCACGGAC	-
PEDV-B3	CAGAATAAACAGCACCACTAG	-
PEDV-FIP	ATGATACCCTCACCTTTAAAGCCTTTTTTTTTATGACTCTGGATGTGTG	Digoxigenin
PEDV-BIP	CCTTACAAATTCTAGCTTTTTGGCATTTTTACATTCTTAAAGGCTAACAACT	-
Probe_PCV2_FITC	TGCAAAATTAGCCCATT	FITC
Probe_PEDV_FITC	GAATCAGATGTGTAATAAAC	FITC

**Table 2:** Calculation of diagnostic sensitivity, diagnostic specificity, accuracy and coefficient of variation (CV) of duplex LAMP-LFD against conventional PCR-based AGE.

		PCR-Based AGE	
		Positive	Negative
Duplex LAMP-LFD	Positive	True positive (TP)	False positive (FP)
	Negative	False negative (FN)	True negative (TN)

Diagnostic sensitivity = TP/TP+FN; Diagnostic specificity = TN/TN + FP; Accuracy = TP + TN/TP + TN + FP + FN.

**Table 3:** Preliminary validation in terms of diagnostic specificity, diagnostic sensitivity, accuracy, coefficient of variation (CV) and process time of PCR-based AGE, LAMP-based AGE and duplex LAMP-LFD for detection of PEDV and PCV2 using 50 swine clinical samples.

Diagnosis Method	True Pedv Positive	True PCV2 Positive	True Negative	Diagnostic Sensitivity (%)	Diagnostic Specificity (%)	Accuracy (%)	Total Time of Detection
PCR-based AGE	16	14	20	100	100	100	2.5 h
LAMP-based AGE	16	14	20	100	100	100	2 h
Duplex LAMP-LFD	16	14	20	100	100	100	1.5 h

**Table 4:** Comparison of molecular methods for detection of PEDV and/or PCV2.

Viruses	Methods	Samples	Limit of Detection	Analytical Specificity	% Diagnostic Sensitivity	% Diagnostic Specificity	% Accuracy	% Sensitivity References
PEDV, PCV2	Duplex-LAMP-LFD	Clinical specimens	0.1 ng/μL 0.246 ng/µL	No cross-reaction with FMDV, ADV, TGEV, PRRSV (EU strain and US strain), CSFV, SIV.	100%	100%	100%	This study
PEDV, TGEV, PRV-A, PKV, PsaV, PDCoV	Multiplex RT-PCR	Feces	10^0^–10^1^ ng cDNA of each virus	No cross-reaction with any other major viruses in swine.	N/A*	N/A*	N/A*	[30]
PEDV, PDCoV, PToV, SADS-CoV	*Taq* Man-probe-based multiplex real-time PCR	Feces	1 × 10^2^ copies/μL	No cross-reaction with TGEV, PoRV, PSV, PTV, CSFV, PKV.	N/A*	N/A*	N/A*	[35]
PEDV, TGEV	Dual ultrasensitive nanoparticle DNA probe-based PCR assay (dual UNDP-PCR)	Feces	25 copies/g	No cross-reaction with PPV, PRV, CSFV, PCV2, PRRSV.	N/A*	N/A*	N/A*	[34]
PEDV	RT-LAMP combined with hydroxynaphthol blue metal indicator	Feces, small intestine	50 RNA copies per reaction	No cross-reaction with TGEV, PDCoV, PRV, type 1 and 2 PRRSVs, CSFV, PCV2, PPV, SIV.	N/A*	N/A*	N/A*	[32]
PEDV, PDCoV, SADS-CoV	Microfluidic-RT-LAMP chip	Feces, intestinal contents	10^1^ copies/μL, 10^2^ copies/μL 10^2^ copies/μL	No cross-reaction with CSFV, PPV, JEV, PCV2, PRRSV, PRV, SIV, FMDV, SVV, RV, TGEV.	92.24%, 92.19% 91.23%	100% 100% 100%	N/A*	[41]
PEDV	Droplet digital PCR	Small intestine, feces, serum	0.26 copies/μL	No cross-reaction with PRV, PRRSV, PDCoV, CSFV, TGEV, PRCV, SADS-CoV, *Actinobacillus pleuropneumoniae, Haemophilus parasuis,* *Streptococcus suis*, *Escherichia coli*, *Salmonella typhimurium, Clostridium* (*Clostridioides*) *difficile, Clostridium perfringens.*	98.6%	100%	N/A*	[29]
PEDV, TGEV, CSFV, PRRSV	Multiplex RT-PCR assay	Clinical specimens	1 × 10^5^ copies 1 × 10^3^ copies 1 × 10^3^ copies 1 × 10^3^ copies	No cross-reaction with PCV2, BVDV, RSV, H5N1.	N/A*	N/A*	N/A*	[40]
PEDV	One-step real-time RT-PCR	Spiked Feces, Spiked jejunum matrices	50 copies/5 μL 100 copies/5 μl	No cross-reaction with PRCV, IBV, TCoV, PRRSV, CSFV, ASFV, SIV-H1N1, SIV-H1N2, SIV-H3N2, H3N2, PCV2.	100%	100%	N/A*	[28]
PEDV	DNA Barcode-Based Aptasensor	Saliva	0.37 μg/mL	N/A	83%	100%	N/A*	[38]
PEDV, TGEV, PRV-A, PDCoV, SADS-CoV	Dual priming oligonucleotide system-based multiplex RT-PCR assay	Intestinal samples	10^3^–10^4^ copies/μL plasmid of each virus	No cross-reaction with PRRSV, APPV, SVV, PCV3, PRCV, PTV, PPV.	100% coincidence rate with that of the RT-PCR method in the evaluation of 181 swine intestinal samples.			[37]
PCV2	LAMP Coupled CRISPR-Cas12a Module	Blood	1 copy/μL	No cross-reaction with PCV1, PCV3, PEDV, CSFV, PRRSV.	100% coincidence rate with that of the quantitative PCR (qPCR) method in the evaluation of 30 clinical blood samples.			[33]
PCV2	LAMP-LFD	DNA	10 fg	CSFV, Erysipelothrix rhusiopathiae, PRRSV type 1, PRRSV type 2, PCV3, SIV-H1N1, SIV-H1N2, and SIV-H3N2.	N/A*	N/A*	N/A*	[31]
PCV2a, PCV2b	LAMP	Clinical samples	10^3^ copies/reaction	No cross-reaction with PCV1, PPV, PRV, PRRSV.	97.7%	100%	98.2%	[36]
PCV2	LAMP-SYBR Green I Dye	Clinical samples	1 copy	No cross-reaction with PCV, PRV and PPV.	100%	86.96%	89.66%	[42]
PCV2, PRRSV, PRV, PPV, FMDV	Centrifugal microfluidic disk (CMFD) using LAMP	Clinical samples	3.2 × 10^2^ copies per reaction	No cross-reaction with TGEV, PEDV, PoRV.	94.0% coincidence rate with PCR in the evaluation of 232 clinical samples.			[39]

Note: porcine epidemic diarrhea virus (PEDV), porcine circovirus 2 (PCV2), Aujeszky’s disease virus (ADV), transmissible gastroenteritis virus (TGEV), porcine rotavirus A (PRV-A), porcine kobuvirus (PKV), porcine sapovirus (PSaV), porcine deltacoronavirus (PDCoV), porcine torovirus (PToV), swine acute diarrhea syndrome coronavirus (SADS-CoV), classical swine fever virus (CSFV), porcine sapelovirus (PSV), porcine teschenvirus (PTV), porcine rotavirus (PoRV), porcine parvovirus (PPV), pseudorabies virus (PRV), porcine reproductive and respiratory syndrome virus (PRRSV), Japanese encephalitis virus (JEV), swine influenza virus (SIV), foot and mouth disease virus (FMDV), rotavirus (RV), porcine respiratory coronavirus (PRCV), bovine viral diarrhea virus (BVDV), respiratory syncytial virus (RSV), chicken influenza A virus H5N1, infectious bronchitis virus (IBV), turkey coronavirus (TCoV), African swine fever virus (ASFV), atypical porcine pestivirus (APPV), Seneca Valley virus (SVV), porcine circovirus 3 (PCV3), porcine teschovirus (PTV). * N/A represents not applicable.

## Data Availability

Not applicable.

## References

[1] Jung K., Saif L.J. (2015). Porcine epidemic diarrhea virus infection: Etiology, epidemiology, pathogenesis and immunoprophylaxis. Vet. J..

[2] Jung K., Saif L.J., Wang Q. (2020). Porcine epidemic diarrhea virus (PEDV): An update on etiology, transmission, pathogenesis, and prevention and control. Virus Res..

[3] Calsamiglia M., Segales J., Quintana J., Rosell C., Domingo M. (2002). Detection of porcine circovirus types 1 and 2 in serum and tissue samples of pigs with and without postweaning multisystemic wasting syndrome. J. Clin. Microbiol..

[4] Kocherhans R., Bridgen A., Ackermann M., Tobler K. (2001). Completion of the porcine epidemic diarrhoea coronavirus (PEDV) genome sequence. Virus Genes.

[5] Bosch B.J., van der Zee R., de Haan C.A., Rottier P.J. (2003). The coronavirus spike protein is a class I virus fusion protein: Structural and functional characterization of the fusion core complex. J. Virol..

[6] Liu Q., Wang H.Y. (2021). Porcine enteric coronaviruses: An updated overview of the pathogenesis, prevalence, and diagnosis. Vet. Res. Commun..

[7] Li R., Tian X., Pang J., Li L., Yuan J., Tian Z., Wang Z. (2022). Point-of-Care Tests for Rapid Detection of Porcine Epidemic Diarrhea Virus: A Systematic Review and Meta-Analysis. Viruses.

[8] Rose N., Opriessnig T., Grasland B., Jestin A. (2012). Epidemiology and transmission of porcine circovirus type 2 (PCV2). Virus Res..

[9] Allan G.M., McNeilly F., Kennedy S., Daft B., Clarke E.G., Ellis J.A., Haines D.M., Meehan B.M., Adair B.M. (1998). Isolation of porcine circovirus-like viruses from pigs with a wasting disease in the USA and Europe. J. Vet. Diagn. Invest..

[10] Ellis J., Hassard L., Clark E., Harding J., Allan G., Willson P., Strokappe J., Martin K., McNeilly F., Meehan B. (1998). Isolation of circovirus from lesions of pigs with postweaning multisystemic wasting syndrome. Can. Vet. J..

[11] Madson D.M., Opriessnig T. (2011). Effect of porcine circovirus type 2 (PCV2) infection on reproduction: Disease, vertical transmission, diagnostics and vaccination. Anim. Health Res. Rev..

[12] Patterson A.R., Opriessnig T. (2010). Epidemiology and horizontal transmission of porcine circovirus type 2 (PCV2). Anim. Health Res. Rev..

[13] Franzo G., Segales J. (2020). Porcine Circovirus 2 Genotypes, Immunity and Vaccines: Multiple Genotypes but One Single Serotype. Pathogens.

[14] Breitbart M., Delwart E., Rosario K., Segales J., Varsani A., Ictv Report C. (2017). ICTV Virus Taxonomy Profile: Circoviridae. J. Gen. Virol..

[15] Franzo G., Segales J. (2018). Porcine circovirus 2 (PCV-2) genotype update and proposal of a new genotyping methodology. PLoS ONE.

[16] Rudova N., Buttler J., Kovalenko G., Sushko M., Bolotin V., Muzykina L., Zinenko O., Stegniy B., Dunaiev Y., Sytiuk M. (2022). Genetic Diversity of Porcine Circovirus 2 in Wild Boar and Domestic Pigs in Ukraine. Viruses.

[17] Bhattacharjee U., Sen A., Sharma I. (2021). Development of cost-effective quantitative PCR method for parallel detection of porcine circovirus2 and porcine parvovirus in perspective of North-eastern India. Trop. Anim. Health Prod..

[18] Brunborg I.M., Moldal T., Jonassen C.M. (2004). Quantitation of porcine circovirus type 2 isolated from serum/plasma and tissue samples of healthy pigs and pigs with postweaning multisystemic wasting syndrome using a TaqMan-based real-time PCR. J. Virol. Methods.

[19] Kim J., Chae C. (2002). Double in situ hybridization for simultaneous detection and differentiation of porcine circovirus 1 and 2 in pigs with postweaning multisystemic wasting syndrome. Vet. J..

[20] Liu Y.B., Zhang L., Xue Q.H., Ning Y.B., Zhang Z.G. (2011). Development of a loop-mediated isothermal amplification assay for porcine circovirus type 2. Virol. Sin..

[21] Racine S., Kheyar A., Gagnon C.A., Charbonneau B., Dea S. (2004). Eucaryotic expression of the nucleocapsid protein gene of porcine circovirus type 2 and use of the protein in an indirect immunofluorescence assay for serological diagnosis of postweaning multisystemic wasting syndrome in pigs. Clin. Diagn. Lab. Immunol..

[22] Segales J. (2012). Porcine circovirus type 2 (PCV2) infections: Clinical signs, pathology and laboratory diagnosis. Virus Res..

[23] Sun S.Q., Guo H.C., Sun D.H., Yin S.H., Shang Y.J., Cai X.P., Liu X.T. (2010). Development and validation of an ELISA using a protein encoded by ORF2 antigenic domain of porcine circovirus type 2. Virol. J..

[24] Szczotka A., Stadejek T., Pejsak Z. (2011). A comparison of immunohistochemistry and in situ hybridization for the detection of porcine circovirus type 2 in pigs. Pol. J. Vet. Sci..

[25] Jung K., Ha Y., Ha S.K., Kim J., Choi C., Park H.K., Kim S.H., Chae C. (2006). Identification of porcine circovirus type 2 in retrospective cases of pigs naturally infected with porcine epidemic diarrhoea virus. Vet. J..

[26] Ouyang T., Zhang X., Liu X., Ren L. (2019). Co-Infection of Swine with Porcine Circovirus Type 2 and Other Swine Viruses. Viruses.

[27] Nguyen T.T., Kwon H.J., Kim I.H., Hong S.M., Seong W.J., Jang J.W., Kim J.H. (2013). Multiplex nested RT-PCR for detecting avian influenza virus, infectious bronchitis virus and Newcastle disease virus. J. Virol. Methods.

[28] Bigault L., Brown P., Bernard C., Blanchard Y., Grasland B. (2020). Porcine epidemic diarrhea virus: Viral RNA detection and quantification using a validated one-step real time RT-PCR. J. Virol. Methods.

[29] Cao W.W., He D.S., Chen Z.J., Zuo Y.Z., Chen X., Chang Y.L., Zhang Z.G., Ye L., Shi L. (2020). Development of a droplet digital PCR for detection and quantification of porcine epidemic diarrhea virus. J. Vet. Diagn. Invest..

[30] Ding G., Fu Y., Li B., Chen J., Wang J., Yin B., Sha W., Liu G. (2020). Development of a multiplex RT-PCR for the detection of major diarrhoeal viruses in pig herds in China. Transbound. Emerg. Dis..

[31] Jang M., Kim S., Song J., Kim S. (2021). Highly sensitive and rapid detection of porcine circovirus 2 by avidin-biotin complex based lateral flow assay coupled to isothermal amplification. Anal. Methods.

[32] Kim J.K., Kim H.R., Kim D.Y., Kim J.M., Kwon N.Y., Park J.H., Park J.Y., Kim S.H., Lee K.K., Lee C. (2021). A simple colorimetric detection of porcine epidemic diarrhea virus by reverse transcription loop-mediated isothermal amplification assay using hydroxynaphthol blue metal indicator. J. Virol. Methods.

[33] Lei L., Liao F., Tan L., Duan D., Zhan Y., Wang N., Wang Y., Peng X., Wang K., Huang X. (2022). LAMP Coupled CRISPR-Cas12a Module for Rapid, Sensitive and Visual Detection of Porcine Circovirus 2. Animals.

[34] Luo L., Chen J., Li X., Qiao D., Wang Z., Wu X., Du Q., Tong D., Huang Y. (2020). Establishment of method for dual simultaneous detection of PEDV and TGEV by combination of magnetic micro-particles and nanoparticles. J. Infect. Chemother..

[35] Pan Z., Lu J., Wang N., He W.T., Zhang L., Zhao W., Su S. (2020). Development of a TaqMan-probe-based multiplex real-time PCR for the simultaneous detection of emerging and reemerging swine coronaviruses. Virulence.

[36] Qiu X., Li T., Zhang G., Cao J., Jin Y., Xing G., Liao M., Zhou J. (2012). Development of a loop-mediated isothermal amplification method to rapidly detect porcine circovirus genotypes 2a and 2b. Virol. J..

[37] Si G., Niu J., Zhou X., Xie Y., Chen Z., Li G., Chen R., He D. (2021). Use of dual priming oligonucleotide system-based multiplex RT-PCR assay to detect five diarrhea viruses in pig herds in South China. AMB Express.

[38] Victorious A., Zhang Z., Chang D., Maclachlan R., Pandey R., Xia J., Gu J., Hoare T., Soleymani L., Li Y. (2022). A DNA Barcode-Based Aptasensor Enables Rapid Testing of Porcine Epidemic Diarrhea Viruses in Swine Saliva Using Electrochemical Readout. Angew. Chem. Int. Ed. Engl..

[39] Yuan X., Lv J., Lin X., Zhang C., Deng J., Wang C., Fan X., Wang Y., Xu H., Wu S. (2019). Multiplex detection of six swine viruses on an integrated centrifugal disk using loop-mediated isothermal amplification. J. Vet. Diagn. Invest..

[40] Zhao Y., Liu F., Li Q., Wu M., Lei L., Pan Z. (2019). A multiplex RT-PCR assay for rapid and simultaneous detection of four RNA viruses in swine. J. Virol. Methods.

[41] Zhou L., Chen Y., Fang X., Liu Y., Du M., Lu X., Li Q., Sun Y., Ma J., Lan T. (2020). Microfluidic-RT-LAMP chip for the point-of-care detection of emerging and re-emerging enteric coronaviruses in swine. Anal. Chim. Acta.

[42] Zhou S., Han S., Shi J., Wu J., Yuan X., Cong X., Xu S., Wu X., Li J., Wang J. (2011). Loop-mediated isothermal amplification for detection of porcine circovirus type 2. Virol. J..

